# International Winter Wheat nurseries data: Facultative and Winter Wheat Observation Nurseries and International Winter Wheat yield trials for semi-arid and irrigated conditions

**DOI:** 10.1016/j.dib.2022.107902

**Published:** 2022-02-02

**Authors:** Mesut Keser, Beyhan Akin, Fatih Ozdemir, Pietro Bartolini, Asma Jeitani

**Affiliations:** a International Center for Agricultural Research in the Dry Areas (ICARDA), Ankara, Turkey; b International Maize and Wheat Improvement Center (CIMMYT), Izmir, Turkey; c Bahri Dagdas International Agricultural Research Institute (BDIARI), Konya, Turkey; d International Center for Agricultural Research in the Dry Areas (ICARDA), Florence, Italy; e International Center for Agricultural Research in the Dry Areas (ICARDA), Beirut, Lebanon

**Keywords:** Winter wheat, Breeding, Germplasm exchange, International nurseries, Multi-locations

## Abstract

This data paper describes the content of 16 datasets collected under the International Winter Wheat Improvement Program (IWWIP), an alliance between Turkey-CIMMYT-ICARDA (TCI), during the 2015–2016, 2016–2017, 2017–2018 and 2018–2019 seasons. Data was collected from the Facultative and Winter Wheat Observation Nursery (FAWWON) and the International Winter Wheat Yield Trials (IWWYT) conducted under semi-arid and irrigated conditions across different countries. Data on all nurseries was collected during the growing season by IWWIP's team and cooperators in their local environments. It was compiled at the end of the wheat season by IWWIP's team. Multi-locational data can be used to select advanced lines that fit to collaborators’ growing environment. The selected germplasm can either be used as a parent in their breeding programs or be released as a variety in their country.

## Specifications Table

**Table:** 

Subject	Agronomy and Crop Science
Specific subject area	Winter wheat improvement, germplasm exchange
Type of data	Tables Biplots
How data were acquired	• Data for genotypes/advanced lines in multi-locations, yields, disease screening and traits were collected by IWWIP's team and local cooperators through field trials and laboratory screenings. • The Molecular Markers study was outsourced to a private company. • The yield and ranking biplots were made using Genstat software.
Data format	Tables in CSV files Biplots in PNG and Pdf files
Parameters for data collection	Parameters for data collection are not always standardized among the cooperators, particularly field measurements and observation data, as each cooperator might use different evaluation scales or data collection methods. All relevant details are described thoroughly in the CSV file “DataDictionary_ElementDescription” of each dataset.
Description of data collection	The data was collected by IWWIP's team and cooperators at local level each with their personnel and labor organization. Data compilation was performed by IWWIP's team.
Data source location	The data was collected by IWWIP's team and cooperators at local level. A detailed list of the cooperators involved in the experiment is provided in the CSV file “Cooperators” of each dataset. The file also includes the coordinates of each research station. The cooperators operate in the following countries: • Afghanistan • Algeria • Armenia • Austria • Azerbaijan • Bulgaria • Canada • Croatia • France • Georgia • Germany • Hungary • Iran • Italy • Kazakhstan • Kenya • Lebanon • Lithuania • Morocco • Pakistan • Romania • Russia • Serbia • South Africa • Spain • Switzerland • Tajikistan • Turkmenistan • Turkey • Ukraine • Uzbekistan
Data accessibility	All 16 datasets are available as open access files on MEL dataverse. Season 2016 18th International Winter Wheat Trial - Semi Arid condition (18IWWYT-SA): https://hdl.handle.net/20.500.11766.1/FK2/QHBKSK 19th International Winter Wheat Yield Trial - Irrigation condition (19IWWYT-IRR): https://hdl.handle.net/20.500.11766.1/FK2/QWBP59 23rd Facultative Winter Wheat Observation Nurseries - Semi Arid condition (23FAWWON-SA): https://hdl.handle.net/20.500.11766.1/FK2/7027BL 23rd Facultative Winter Wheat Observation Nurseries - Irrigation condition (23FAWWON-IRR): https://hdl.handle.net/20.500.11766.1/FK2/SOQZIZ Season 2017 19th International Winter Wheat Trial - Semi Arid condition (19IWWYT-SA): https://hdl.handle.net/20.500.11766.1/FK2/OEK62N 20th International Winter Wheat Yield Trial - Irrigation condition (20IWWYT-IRR): https://hdl.handle.net/20.500.11766.1/FK2/ROI1XB 24th Facultative Winter Wheat Observation Nurseries - Semi Arid condition (24FAWWON-SA): https://hdl.handle.net/20.500.11766.1/FK2/IDX1ZA 24th Facultative Winter Wheat Observation Nurseries - Irrigation condition (24FAWWON-IRR): https://hdl.handle.net/20.500.11766.1/FK2/2S1RLL Season 2018 20th International Winter Wheat Trial - Semi Arid condition (20IWWYT-SA): https://hdl.handle.net/20.500.11766.1/FK2/6513PJ 21st International Winter Wheat Yield Trial - Irrigation condition (21IWWYT-IRR): https://hdl.handle.net/20.500.11766.1/FK2/1TNRPD 25th Facultative Winter Wheat Observation Nurseries - Semi Arid condition (25FAWWON-SA): https://hdl.handle.net/20.500.11766.1/FK2/J63QUK 25th Facultative Winter Wheat Observation Nurseries - Irrigation condition (25FAWWON-IRR): https://hdl.handle.net/20.500.11766.1/FK2/EB7YDS Season 2019 21st International Winter Wheat Trial - Semi Arid condition (21IWWYT-SA): https://hdl.handle.net/20.500.11766.1/FK2/RPERGO 22nd International Winter Wheat Yield Trial - Irrigation condition (22IWWYT-IRR): https://hdl.handle.net/20.500.11766.1/FK2/10UCHP 26th Facultative Winter Wheat Observation Nurseries - Semi Arid condition (26FAWWON-SA): https://hdl.handle.net/20.500.11766.1/FK2/VCOACL 26th Facultative Winter Wheat Observation Nurseries - Irrigation condition (26FAWWON-IRR): https://hdl.handle.net/20.500.11766.1/FK2/L6SRZN

## Value of the Data

• The experiments are conducted in multiple locations and in diverse growing environments providing information on the performance of the genotypes tested in those environments including disease resistance, quality attributes and molecular data in some years. The collected data is free for use by breeding programs and breeders for their evaluations and decisions on genotypes.
• The breeding and research programs, which are cooperators in IWWIP and obtain germplasm from IWWIP, benefit greatly through access to the data on germplasm that has been generated over diverse regions and environments.
• In general, the observation nurseries are targeted to identify adapted genotypes that can be potentially used as parents in the breeding programs of the corresponding NARS, while the yield trials are primarily distributed to identify widely adapted genotypes to be released in the corresponding countries and to be used in future crosses at IWWIP. Multi-locational data can be used to select advanced lines that fit to their growing environment. The selected germplasm can be used as a parent in breeding programs or be released as a variety in the corresponding country.

## 1 Data Description

This article describes 16 datasets collected under the International Winter Wheat Improvement Program (IWWIP), an alliance between Turkey-CIMMYT-ICARDA (TCI), during the 2015–2016, 2016–2017, 2017–2018 and 2018–2019 seasons.

Datasets FAWWON-SA and FAWWON-IRR contain the data collected from the Facultative and Winter Wheat Observation Nursery (FAWWON) trials implemented under semi-arid and under irrigated conditions, respectively.

**Fig. 1:**
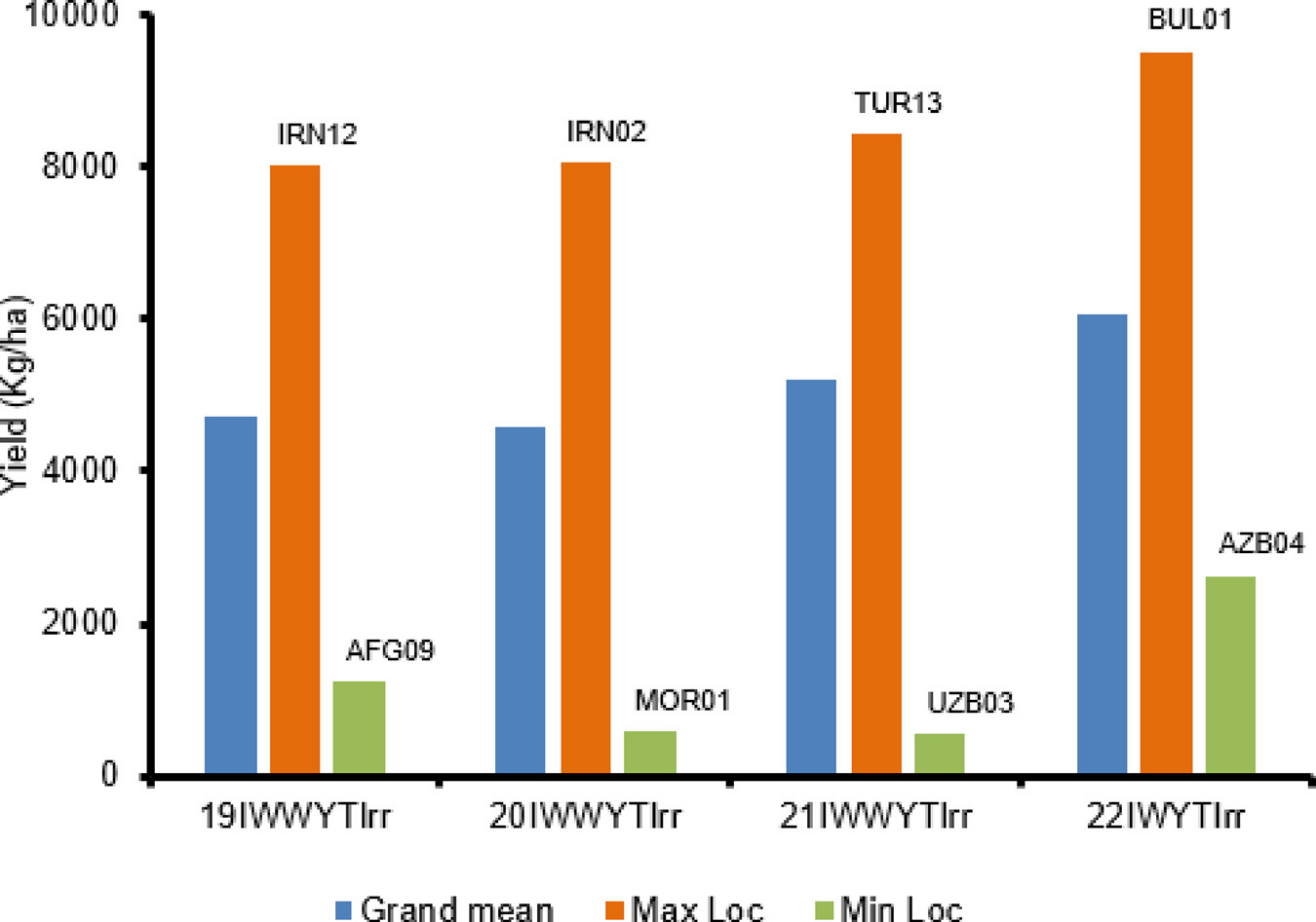
Grand means of International Winter Wheat Yield Trials under irrigated conditions in a year and highest and lowest location mean yield in the same year.

**Fig. 2:**
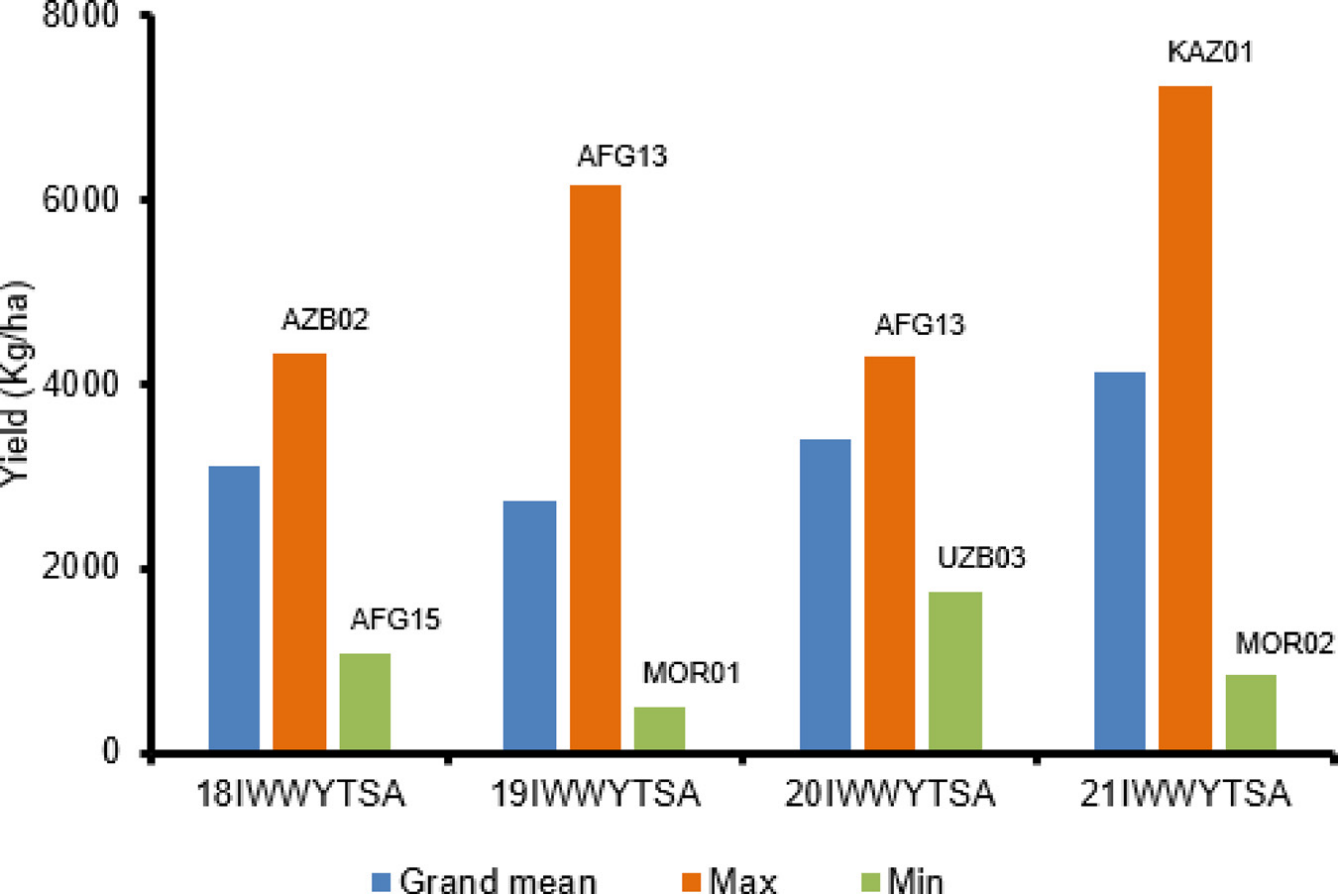
Grand means of International Winter Wheat Yield Trials under semiarid conditions in a year and highest and lowest location mean yield in the same year.

Datasets IWWYT-SA and IWWYT-IRR contain the data collected from the International Winter Wheat Yield Trial (IWWYT) trials conducted under semi-arid and under irrigated conditions, respectively (Figs. 1 and 2).

The link provided with each dataset (referring to the specification table) redirects the user to a series of data files. These are presented either in CSV format (in the case of tables), or PNG and Pdf formats (in the case of biplots). A summary of the data files content is found in Table 1.

**Table 1:** Summary of data files.

Data File Name	Data File Format	File Variables	File General Description
Data Dictionary: Introduction	CSV	Description, Summary, Start Date, End Date, Authors, Co-authors.	Background explanatory data about the dataset.
Data Dictionary: Element Description	CSV	Element_DisplayName, Description, Unit, Data Type etc.	Explanation of each variable/column and any code used inside the dataset.
Data Dictionary: Unique Identifier	CSV	Element_DisplayName, Unique_Identifier, Source etc.	Reference links to an online resource for elements, terms, and concepts used in the dataset.
Cooperators	CSV	Country, Institution, Geographical coordinates etc.	Data about the cooperators involved in the experiment.
Cultivars	CSV	Name of the nursery, Accession number etc.	General data about the cultivars.
Selected	CSV	Selection results associated to the cooperators code	Data about the selection results.
Heading Days	CSV	Days to Heading, Days to maturity	Data about days to heading and days to maturity collected in each trial site.
Plant Height	CSV	Plant height	Data about plant height measured in each trial site.
Yield	CSV	Yield	Yield data gathered from each trial site.
Diseases	CSV	Yellow Rust, Leaf Rust, Stem Rust, Septoria, Powdery Mildew etc.	Data on disease susceptibility.
Miscellaneous	CSV	Test Weight, Thousand Kernel Weight, Winter Kill, Lodging etc.	Data on other miscellaneous traits collected only by a limited number of cooperators.
Quality	CSV	Kernel weight, Kernel diameter, Kernel Moisture, Gluten peak maximum time, Gluten aggregation energy, Water absorption etc.	Data on genotype quality evaluation. Note: file found only in dataset 20IWWYT-IRR
Molecular Markers	CSV	Rht-B1, Rht-D1_SNP, Ppd-A1, Vrn-B1, Vrn-D1 etc.	The file provides data on different alleles identified using molecular markers. Note: file found only in datasets 25FAWWON-IRR and 25FAWWON-SA
Data with replications	CSV	Days to Heading Replication 1, Days to Heading Replication 2, Lodging Replication 1, Lodging Replication 2 etc.	Measured trials data provided with their replications Note: file found only in dataset 21IWWYT-SA
Biplot	PNG	–	The image is a screenshot of a biplot analysis. Note: file found only in datasets 19IWWYT-SA and 20IWWYT-IRR
Yield Biplot Rank	CSV	Entry, Overall yield Rank, etc.	Variables of the Bi-plot for grain yield across different sites Note: file found only in datasets 21IWWYT-IRR and 20IWWYT-SA
Biplot Trait Association	Pdf	–	This file is linked to the “Yield Biplot Rank” file. Bi-plot traits association based on averages across all locations and disease severity (rusts: SR, LR, YR) at locations with highest infection. Note: file found only in datasets 21IWWYT-IRR and 20IWWYT-SA
Biplot Rank	Pdf	–	This file is linked to the “Yield Biplot Rank” file. Note: file found only in dataset 20IWWYT-SA
GGE Biplot Analysis	Pdf	–	Note: file found only in datasets 21IWWYT-SA and 22IWWYT-IRR
GGE Biplot for yield	Pdf	-	Note: file found only in datasets 18IWWYT-SA and 19IWWYT-IRR

## 2 Experimental Design, Materials and Methods

Based on the yield trial performance, short stature high-yielding germplasm developed by IWWIP and contributed by the cooperators is included into FAWWON for irrigated environments. Whereas entries with resistance to moisture stress are included in FAWWON for semiarid environments. The germplasm in this nursery may have variable height and some tall lines may also be included.

In addition, each year interested breeding programs submit their new cultivars and advanced breeding lines to IWWIP for inclusion into international testing. Once in Turkey, the material goes through quarantine inspection before being planted for primary evaluation on small plots in locations highly conducive to the key diseases. Germplasm with disease resistance and general adaptation traits is multiplied and yield-tested during a second year.

The next step is selecting the elite germplasm proven to perform well in FAWWON to be included in IWWYT. The objective of this nursery is to identify broadly adapted high-yielding germplasm, which can be potentially used as cultivars and parent material [1].

In IWWYT, the genotypes in each trial were planted using alpha lattice design in two replications in a plot size of 5 m length, six rows with 0.2 m spacing between rows. FAWWONs are planted as 1 m two rows with checks every 20.

Data on all nurseries is collected during the growing season by IWWIP's team and cooperators in their local environments. Cooperators use either hardcopy sheets or more recently electronic files. At the end of the wheat season, the data is assembled by IWWIP's team in Ankara in a single spreadsheet and made available on IWWIP's website. The datasets described in this article were curated by ICARDA's Data Management Team, following the standards of the General Dataset Curation Guide (GDCG) [2] and subsequently published through DataverseMEL.

## CRediT authorship contribution statement

**Mesut Keser:** Conceptualization, Methodology, Data curation, Writing – review & editing, Supervision. **Beyhan Akin:** Conceptualization, Methodology, Data curation, Writing – original draft. **Fatih Ozdemir:** Conceptualization, Methodology, Writing – original draft. **Pietro Bartolini:** Data curation, Writing – original draft. **Asma Jeitani:** Data curation, Writing – original draft.

## Declaration of Competing Interest

The authors declare that they have no known competing financial interests or personal relationships which have or could be perceived to have influenced the work reported in this paper.
